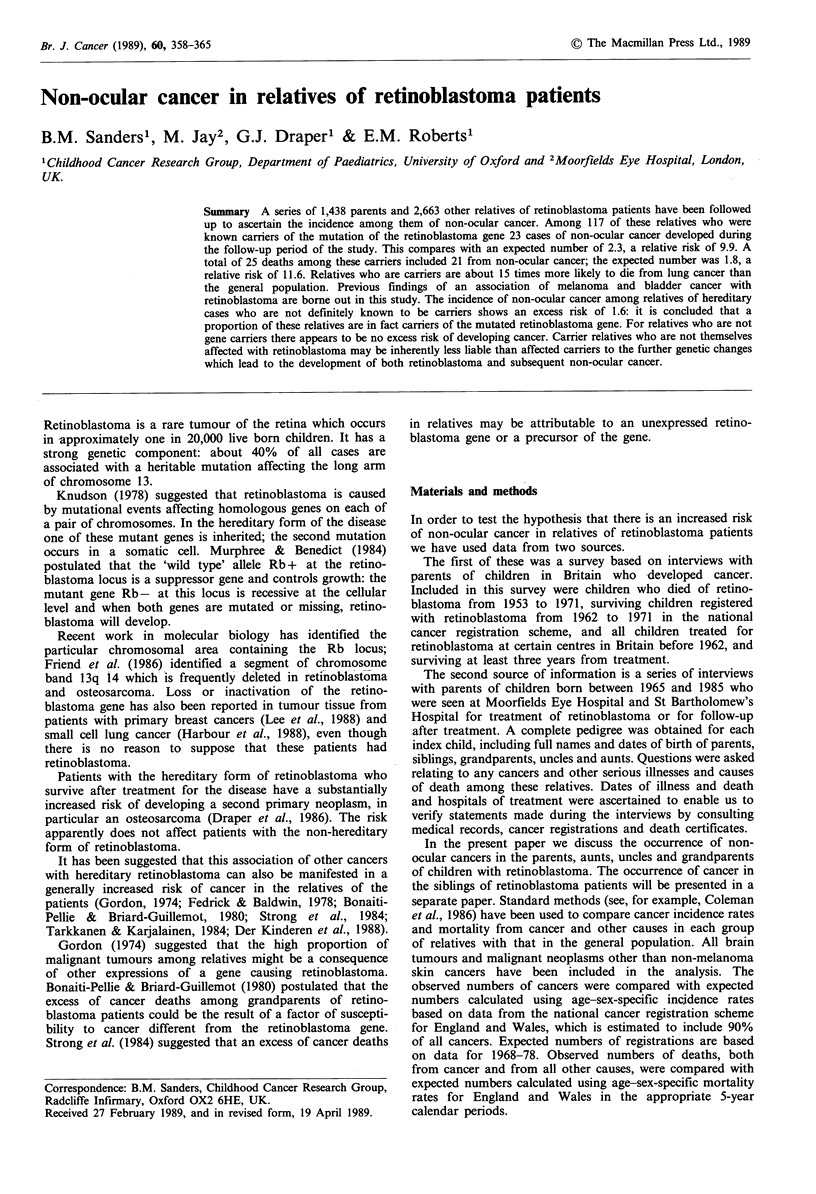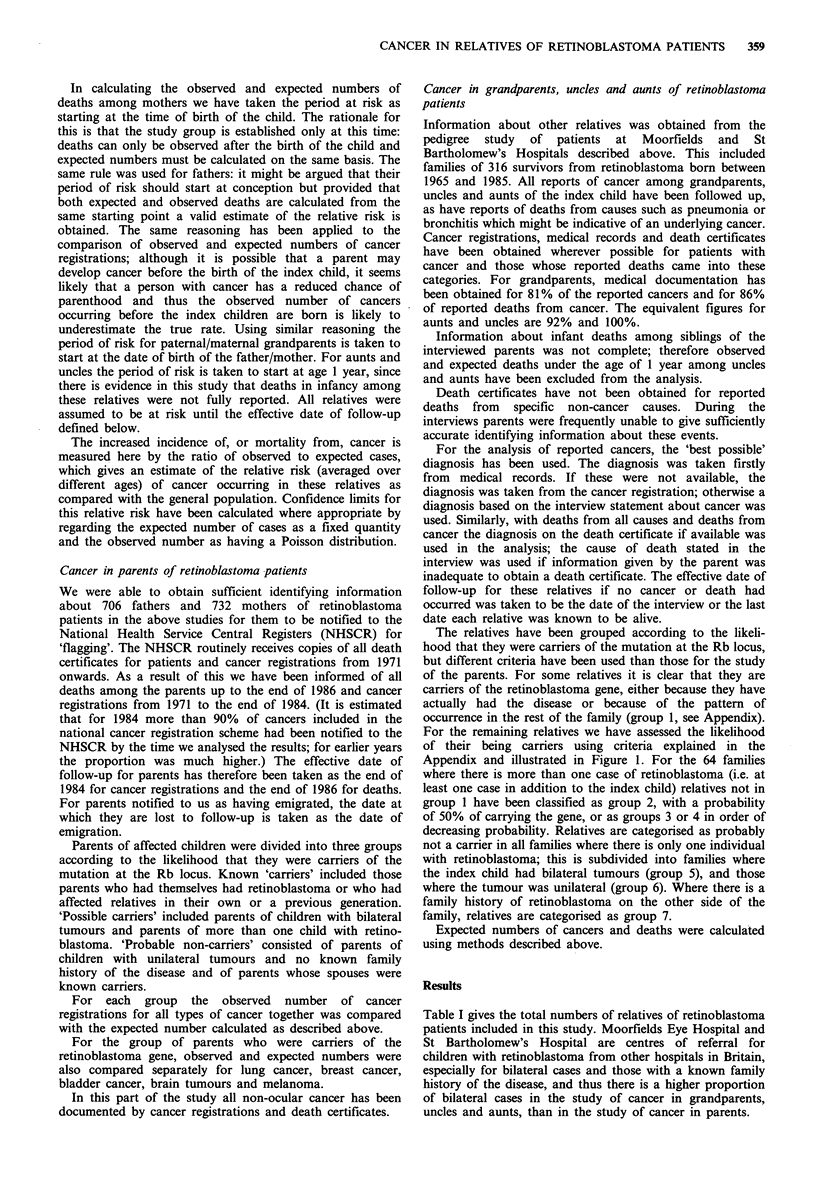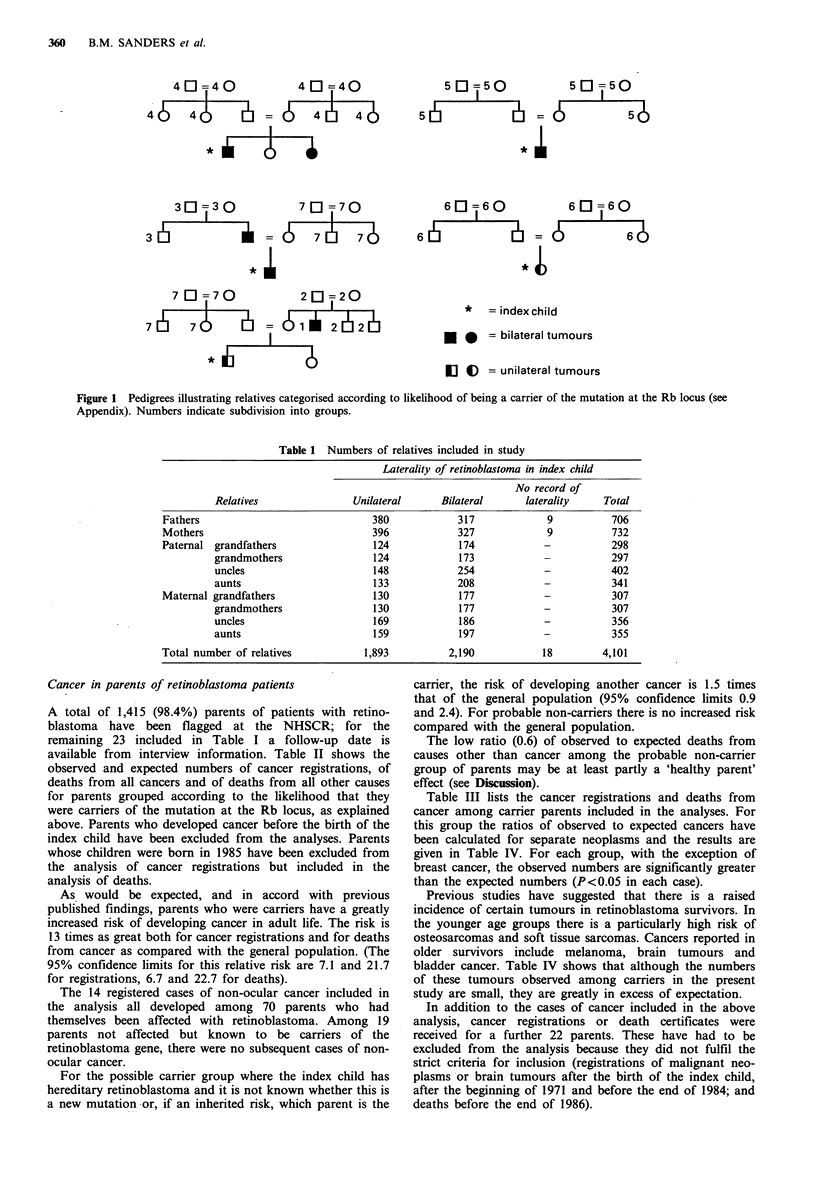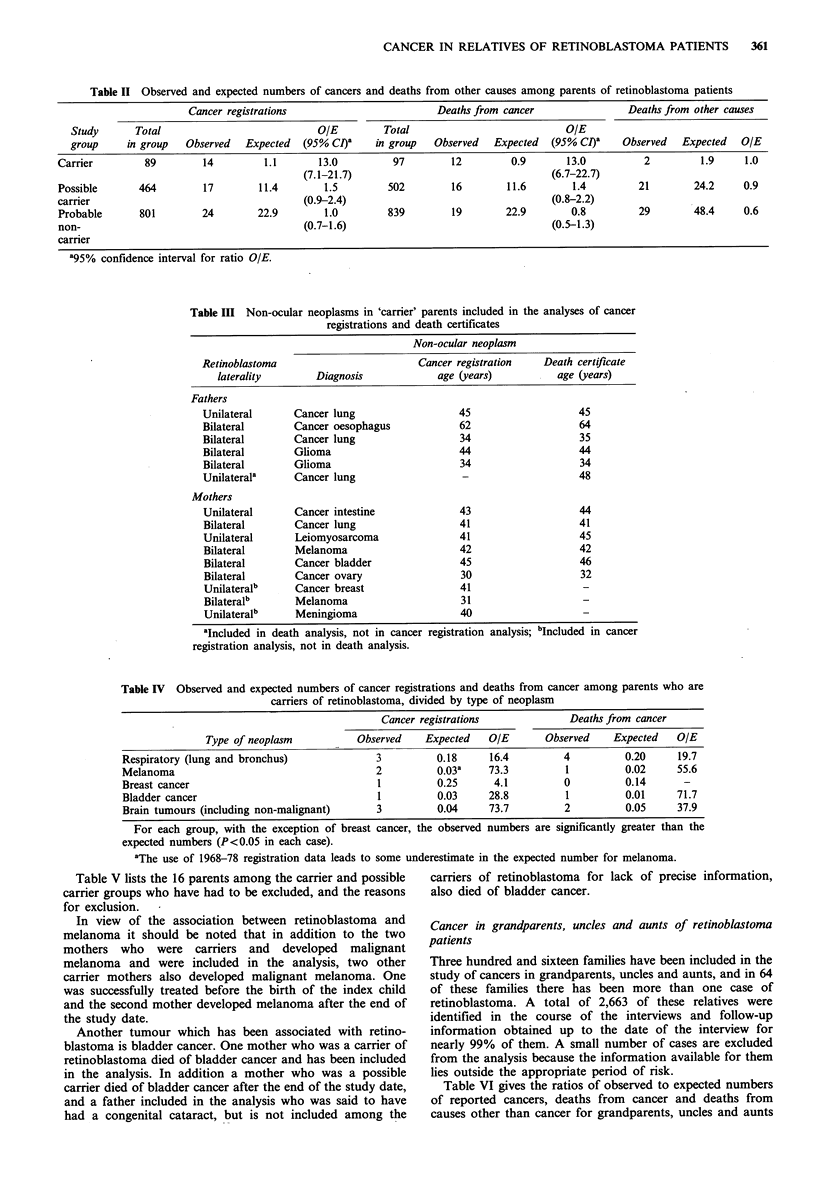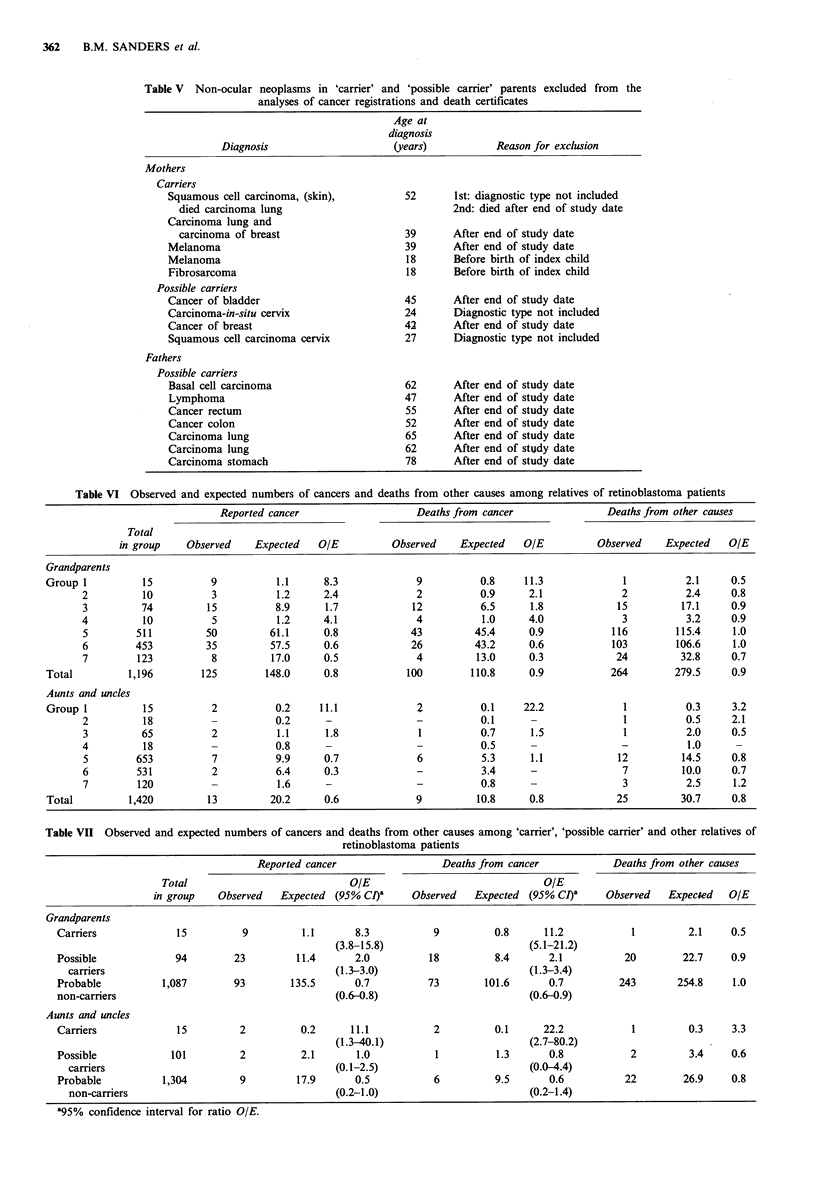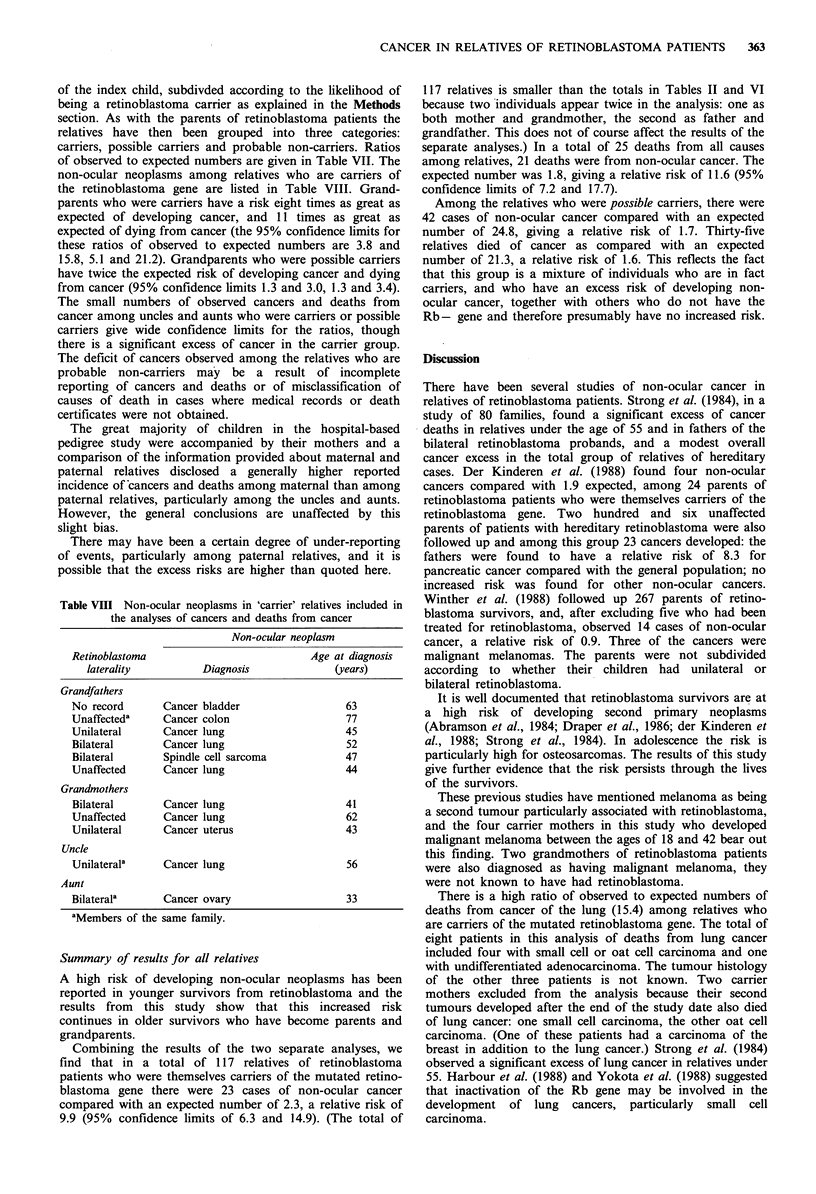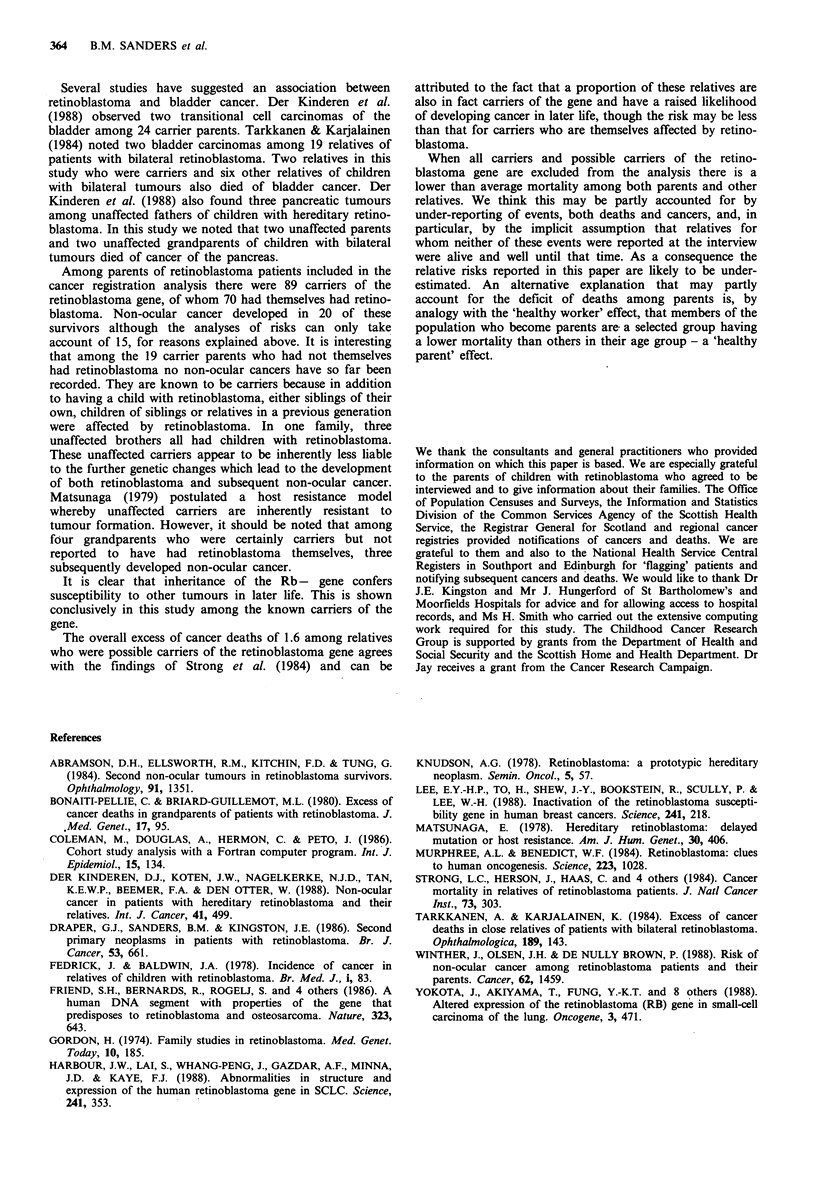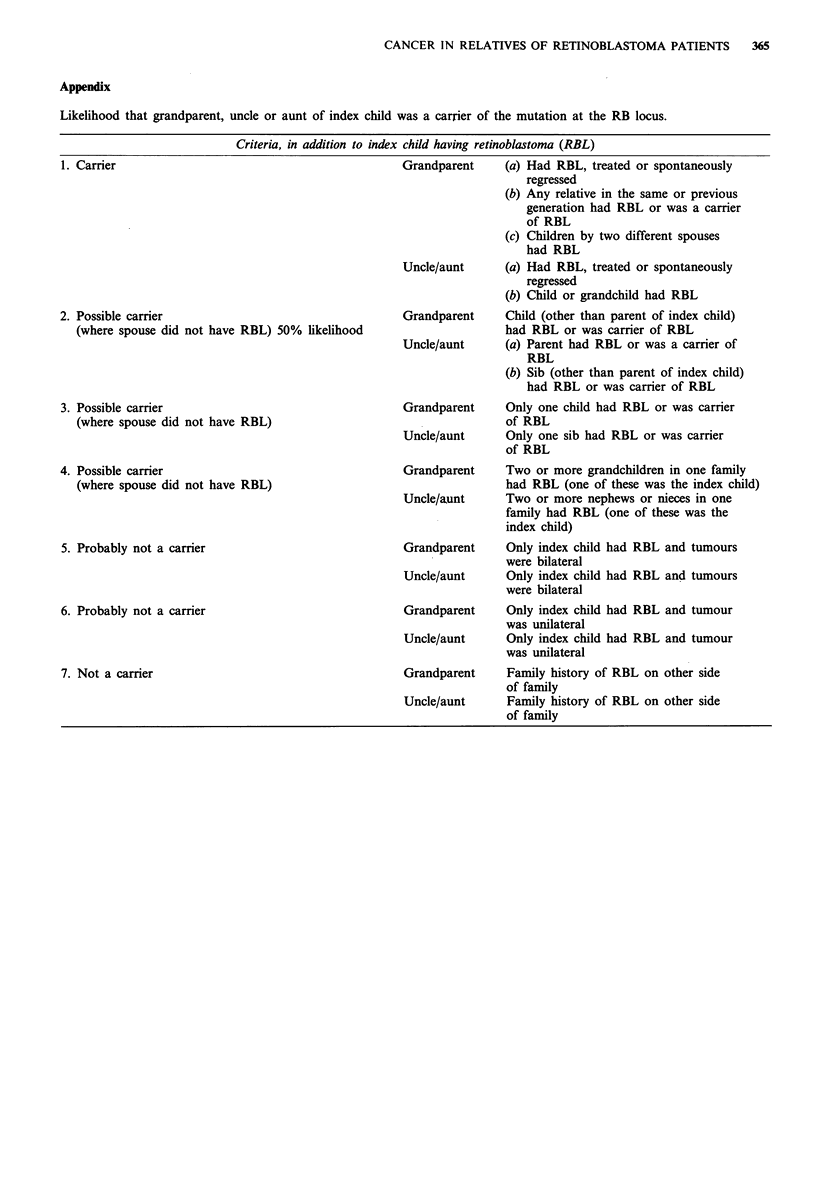# Non-ocular cancer in relatives of retinoblastoma patients.

**DOI:** 10.1038/bjc.1989.285

**Published:** 1989-09

**Authors:** B. M. Sanders, M. Jay, G. J. Draper, E. M. Roberts

**Affiliations:** Department of Paediatrics, University of Oxford, London, UK.

## Abstract

A series of 1,438 parents and 2,663 other relatives of retinoblastoma patients have been followed up to ascertain the incidence among them of non-ocular cancer. Among 117 of these relatives who were known carriers of the mutation of the retinoblastoma gene 23 cases of non-ocular cancer developed during the follow-up period of the study. This compares with an expected number of 2.3, a relative risk of 9.9. A total of 25 deaths among these carriers included 21 from non-ocular cancer; the expected number was 1.8, a relative risk of 11.6. Relatives who are carriers are about 15 times more likely to die from lung cancer than the general population. Previous findings of an association of melanoma and bladder cancer with retinoblastoma are borne out in this study. The incidence of non-ocular cancer among relatives of hereditary cases who are not definitely known to be carriers shows an excess risk of 1.6: it is concluded that a proportion of these relatives are in fact carriers of the mutated retinoblastoma gene. For relatives who are not gene carriers there appears to be no excess risk of developing cancer. Carriers relatives who are not themselves affected with retinoblastoma may be inherently less liable than affected carriers to the further genetic changes which lead to the development of both retinoblastoma and subsequent non-ocular cancer.


*Br. J. Cancer* (1989), **60**, 358–365 © The Macmillan Press Ltd., 1989

### Non-ocular cancer in relatives of retinoblastoma patients

B.M. Sanders[1], M. Jay[2], G.J. Draper[1] & E.M. Roberts[1]

[1]*Childhood Cancer Research Group, Department of Paediatrics, University of Oxford and* [2]*Moorfields Eye Hospital, London, UK.*

**Summary** A series of 1,438 parents and 2,663 other relatives of retinoblastoma patients have been followed up to ascertain the incidence among them of non-ocular cancer. Among 117 of these relatives who were known carriers of the mutation of the retinoblastoma gene 23 cases of non-ocular cancer developed during the follow-up period of the study. This compares with an expected number of 2.3, a relative risk of 9.9. A total of 25 deaths among these carriers included 21 from non-ocular cancer; the expected number was 1.8, a relative risk of 11.6. Relatives who are carriers are about 15 times more likely to die from lung cancer than the general population. Previous findings of an association of melanoma and bladder cancer with retinoblastoma are borne out in this study. The incidence of non-ocular cancer among relatives of hereditary cases who are not definitely known to be carriers shows an excess risk of 1.6: it is concluded that a proportion of these relatives are in fact carriers of the mutated retinoblastoma gene. For relatives who are not gene carriers there appears to be no excess risk of developing cancer. Carrier relatives who are not themselves affected with retinoblastoma may be inherently less liable than affected carriers to the further genetic changes which lead to the development of both retinoblastoma and subsequent non-ocular cancer.

Retinoblastoma is a rare tumour of the retina which occurs in approximately one in 20,000 live born children. It has a strong genetic component: about 40% of all cases are associated with a heritable mutation affecting the long arm of chromosome 13.

Knudson (1978) suggested that retinoblastoma is caused by mutational events affecting homologous genes on each of a pair of chromosomes. In the hereditary form of the disease one of these mutant genes is inherited; the second mutation occurs in a somatic cell. Murphree & Benedict (1984) postulated that the 'wild type' allele Rb+ at the retinoblastoma locus is a suppressor gene and controls growth: the mutant gene Rb− at this locus is recessive at the cellular level and when both genes are mutated or missing, retinoblastoma will develop.

Recent work in molecular biology has identified the particular chromosomal area containing the Rb locus; Friend *et al.* (1986) identified a segment of chromosome band 13q 14 which is frequently deleted in retinoblastoma and osteosarcoma. Loss or inactivation of the retinoblastoma gene has also been reported in tumour tissue from patients with primary breast cancers (Lee *et al.*, 1988) and small cell lung cancer (Harbour *et al.*, 1988), even though there is no reason to suppose that these patients had retinoblastoma.

Patients with the hereditary form of retinoblastoma who survive after treatment for the disease have a substantially increased risk of developing a second primary neoplasm, in particular an osteosarcoma (Draper *et al.*, 1986). The risk apparently does not affect patients with the non-hereditary form of retinoblastoma.

It has been suggested that this association of other cancers with hereditary retinoblastoma can also be manifested in a generally increased risk of cancer in the relatives of the patients (Gordon, 1974; Fedrick & Baldwin, 1978; Bonaiti-Pellie & Briard-Guillemot, 1980; Strong *et al.*, 1984; Tarkkanen & Karjalainen, 1984; Der Kinderen *et al.*, 1988).

Gordon (1974) suggested that the high proportion of malignant tumours among relatives might be a consequence of other expressions of a gene causing retinoblastoma. Bonaiti-Pellie & Briard-Guillemot (1980) postulated that the excess of cancer deaths among grandparents of retinoblastoma patients could be the result of a factor of susceptibility to cancer different from the retinoblastoma gene. Strong *et al.* (1984) suggested that an excess of cancer deaths in relatives may be attributable to an unexpressed retinoblastoma gene or a precursor of the gene.

Correspondence: B.M. Sanders, Childhood Cancer Research Group, Radcliffe Infirmary, Oxford OX2 6HE, UK.
Received 27 February 1989, and in revised form, 19 April 1989.

### Materials and methods

In order to test the hypothesis that there is an increased risk of non-ocular cancer in relatives of retinoblastoma patients we have used data from two sources.

The first of these was a survey based on interviews with parents of children in Britain who developed cancer. Included in this survey were children who died of retinoblastoma from 1953 to 1971, surviving children registered with retinoblastoma from 1962 to 1971 in the national cancer registration scheme, and all children treated for retinoblastoma at certain centres in Britain before 1962, and surviving at least three years from treatment.

The second source of information is a series of interviews with parents of children born between 1965 and 1985 who were seen at Moorfields Eye Hospital and St Bartholomew's Hospital for treatment of retinoblastoma or for follow-up after treatment. A complete pedigree was obtained for each index child, including full names and dates of birth of parents, siblings, grandparents, uncles and aunts. Questions were asked relating to any cancers and other serious illnesses and causes of death among these relatives. Dates of illness and death and hospitals of treatment were ascertained to enable us to verify statements made during the interviews by consulting medical records, cancer registrations and death certificates.

In the present paper we discuss the occurrence of non-ocular cancers in the parents, aunts, uncles and grandparents of children with retinoblastoma. The occurrence of cancer in the siblings of retinoblastoma patients will be presented in a separate paper. Standard methods (see, for example, Coleman *et al.*, 1986) have been used to compare cancer incidence rates and mortality from cancer and other causes in each group of relatives with that in the general population. All brain tumours and malignant neoplasms other than non-melanoma skin cancers have been included in the analysis. The observed numbers of cancers were compared with expected numbers calculated using age–sex-specific incidence rates based on data from the national cancer registration scheme for England and Wales, which is estimated to include 90% of all cancers. Expected numbers of registrations are based on data for 1968–78. Observed numbers of deaths, both from cancer and from all other causes, were compared with expected numbers calculated using age–sex-specific mortality rates for England and Wales in the appropriate 5-year calendar periods.

In calculating the observed and expected numbers of deaths among mothers we have taken the period at risk as starting at the time of birth of the child. The rationale for this is that the study group is established only at this time: deaths can only be observed after the birth of the child and expected numbers must be calculated on the same basis. The same rule was used for fathers: it might be argued that their period of risk should start at conception but provided that both expected and observed deaths are calculated from the same starting point a valid estimate of the relative risk is obtained. The same reasoning has been applied to the comparison of observed and expected numbers of cancer registrations; although it is possible that a parent may develop cancer before the birth of the index child, it seems likely that a person with cancer has a reduced chance of parenthood and thus the observed number of cancers occurring before the index children are born is likely to underestimate the true rate. Using similar reasoning the period of risk for paternal/maternal grandparents is taken to start at the date of birth of the father/mother. For aunts and uncles the period of risk is taken to start at age 1 year, since there is evidence in this study that deaths in infancy among these relatives were not fully reported. All relatives were assumed to be at risk until the effective date of follow-up defined below.

The increased incidence of, or mortality from, cancer is measured here by the ratio of observed to expected cases, which gives an estimate of the relative risk (averaged over different ages) of cancer occurring in these relatives as compared with the general population. Confidence limits for this relative risk have been calculated where appropriate by regarding the expected number of cases as a fixed quantity and the observed number as having a Poisson distribution.

*Cancer in parents of retinoblastoma patients*

We were able to obtain sufficient identifying information about 706 fathers and 732 mothers of retinoblastoma patients in the above studies for them to be notified to the National Health Service Central Registers (NHSCR) for 'flagging'. The NHSCR routinely receives copies of all death certificates for patients and cancer registrations from 1971 onwards. As a result of this we have been informed of all deaths among the parents up to the end of 1986 and cancer registrations from 1971 to the end of 1984. (It is estimated that for 1984 more than 90% of cancers included in the national cancer registration scheme had been notified to the NHSCR by the time we analysed the results; for earlier years the proportion was much higher.) The effective date of follow-up for parents has therefore been taken as the end of 1984 for cancer registrations and the end of 1986 for deaths. For parents notified to us as having emigrated, the date at which they are lost to follow-up is taken as the date of emigration.

Parents of affected children were divided into three groups according to the likelihood that they were carriers of the mutation at the Rb locus. Known 'carriers' included those parents who had themselves had retinoblastoma or who had affected relatives in their own or a previous generation. 'Possible carriers' included parents of children with bilateral tumours and parents of more than one child with retinoblastoma. 'Probable non-carriers' consisted of parents of children with unilateral tumours and no known family history of the disease and of parents whose spouses were known carriers.

For each group the observed number of cancer registrations for all types of cancer together was compared with the expected number calculated as described above.

For the group of parents who were carriers of the retinoblastoma gene, observed and expected numbers were also compared separately for lung cancer, breast cancer, bladder cancer, brain tumours and melanoma.

In this part of the study all non-ocular cancer has been documented by cancer registrations and death certificates.

*Cancer in grandparents, uncles and aunts of retinoblastoma patients*

Information about other relatives was obtained from the pedigree study of patients at Moorfields and St Bartholomew's Hospitals described above. This included families of 316 survivors from retinoblastoma born between 1965 and 1985. All reports of cancer among grandparents, uncles and aunts of the index child have been followed up, as have reports of deaths from causes such as pneumonia or bronchitis which might be indicative of an underlying cancer. Cancer registrations, medical records and death certificates have been obtained wherever possible for patients with cancer and those whose reported deaths came into these categories. For grandparents, medical documentation has been obtained for 81% of the reported cancers and for 86% of reported deaths from cancer. The equivalent figures for aunts and uncles are 92% and 100%.

Information about infant deaths among siblings of the interviewed parents was not complete; therefore observed and expected deaths under the age of 1 year among uncles and aunts have been excluded from the analysis.

Death certificates have not been obtained for reported deaths from specific non-cancer causes. During the interviews parents were frequently unable to give sufficiently accurate identifying information about these events.

For the analysis of reported cancers, the 'best possible' diagnosis has been used. The diagnosis was taken firstly from medical records. If these were not available, the diagnosis was taken from the cancer registration; otherwise a diagnosis based on the interview statement about cancer was used. Similarly, with deaths from all causes and deaths from cancer the diagnosis on the death certificate if available was used in the analysis; the cause of death stated in the interview was used if information given by the parent was inadequate to obtain a death certificate. The effective date of follow-up for these relatives if no cancer or death had occurred was taken to be the date of the interview or the last date each relative was known to be alive.

The relatives have been grouped according to the likelihood that they were carriers of the mutation at the Rb locus, but different criteria have been used than those for the study of the parents. For some relatives it is clear that they are carriers of the retinoblastoma gene, either because they have actually had the disease or because of the pattern of occurrence in the rest of the family (group 1, see Appendix). For the remaining relatives we have assessed the likelihood of their being carriers using criteria explained in the Appendix and illustrated in Figure 1. For the 64 families where there is more than one case of retinoblastoma (i.e. at least one case in addition to the index child) relatives not in group 1 have been classified as group 2, with a probability of 50% of carrying the gene, or as groups 3 or 4 in order of decreasing probability. Relatives are categorised as probably not a carrier in all families where there is only one individual with retinoblastoma; this is subdivided into families where the index child had bilateral tumours (group 5), and those where the tumour was unilateral (group 6). Where there is a family history of retinoblastoma on the other side of the family, relatives are categorised as group 7.

Expected numbers of cancers and deaths were calculated using methods described above.

### Results

Table I gives the total numbers of relatives of retinoblastoma patients included in this study. Moorfields Eye Hospital and St Bartholomew's Hospital are centres of referral for children with retinoblastoma from other hospitals in Britain, especially for bilateral cases and those with a known family history of the disease, and thus there is a higher proportion of bilateral cases in the study of cancer in grandparents, uncles and aunts, than in the study of cancer in parents.

**Figure 1** Pedigrees illustrating relatives categorised according to likelihood of being a carrier of the mutation at the Rb locus (see Appendix). Numbers indicate subdivision into groups.

**Table 1** Numbers of relatives included in study

| Relatives | | *Laterality of retinoblastoma in index child* | | | |
|---|---|---|---|---|---|
| | | *Unilateral* | *Bilateral* | *No record of laterality* | *Total* |
| Fathers | | 380 | 317 | 9 | 706 |
| Mothers | | 396 | 327 | 9 | 732 |
| Paternal | grandfathers | 124 | 174 | – | 298 |
| | grandmothers | 124 | 173 | – | 297 |
| | uncles | 148 | 254 | – | 402 |
| | aunts | 133 | 208 | – | 341 |
| Maternal | grandfathers | 130 | 177 | – | 307 |
| | grandmothers | 130 | 177 | – | 307 |
| | uncles | 169 | 186 | – | 356 |
| | aunts | 159 | 197 | – | 355 |
| Total number of relatives | | 1,893 | 2,190 | 18 | 4,101 |

*Cancer in parents of retinoblastoma patients*

A total of 1,415 (98.4%) parents of patients with retinoblastoma have been flagged at the NHSCR; for the remaining 23 included in Table I a follow-up date is available from interview information. Table II shows the observed and expected numbers of cancer registrations, of deaths from all cancers and of deaths from all other causes for parents grouped according to the likelihood that they were carriers of the mutation at the Rb locus, as explained above. Parents who developed cancer before the birth of the index child have been excluded from the analyses. Parents whose children were born in 1985 have been excluded from the analysis of cancer registrations but included in the analysis of deaths.

As would be expected, and in accord with previous published findings, parents who were carriers have a greatly increased risk of developing cancer in adult life. The risk is 13 times as great both for cancer registrations and for deaths from cancer as compared with the general population. (The 95% confidence limits for this relative risk are 7.1 and 21.7 for registrations, 6.7 and 22.7 for deaths).

The 14 registered cases of non-ocular cancer included in the analysis all developed among 70 parents who had themselves been affected with retinoblastoma. Among 19 parents not affected but known to be carriers of the retinoblastoma gene, there were no subsequent cases of non-ocular cancer.

For the possible carrier group where the index child has hereditary retinoblastoma and it is not known whether this is a new mutation or, if an inherited risk, which parent is the carrier, the risk of developing another cancer is 1.5 times that of the general population (95% confidence limits 0.9 and 2.4). For probable non-carriers there is no increased risk compared with the general population.

The low ratio (0.6) of observed to expected deaths from causes other than cancer among the probable non-carrier group of parents may be at least partly a 'healthy parent' effect (see **Discussion**).

Table III lists the cancer registrations and deaths from cancer among carrier parents included in the analyses. For this group the ratios of observed to expected cancers have been calculated for separate neoplasms and the results are given in Table IV. For each group, with the exception of breast cancer, the observed numbers are significantly greater than the expected numbers ($P<0.05$ in each case).

Previous studies have suggested that there is a raised incidence of certain tumours in retinoblastoma survivors. In the younger age groups there is a particularly high risk of osteosarcomas and soft tissue sarcomas. Cancers reported in older survivors include melanoma, brain tumours and bladder cancer. Table IV shows that although the numbers of these tumours observed among carriers in the present study are small, they are greatly in excess of expectation.

In addition to the cases of cancer included in the above analysis, cancer registrations or death certificates were received for a further 22 parents. These have had to be excluded from the analysis because they did not fulfil the strict criteria for inclusion (registrations of malignant neoplasms or brain tumours after the birth of the index child, after the beginning of 1971 and before the end of 1984; and deaths before the end of 1986).

**Table II** Observed and expected numbers of cancers and deaths from other causes among parents of retinoblastoma patients

| | *Cancer registrations* | | | | *Deaths from cancer* | | | | *Deaths from other causes* | | |
|---|---|---|---|---|---|---|---|---|---|---|---|
| *Study group* | *Total in group* | *Observed* | *Expected* | *O/E (95% CI)*[a] | *Total in group* | *Observed* | *Expected* | *O/E (95% CI)*[a] | *Observed* | *Expected* | *O/E* |
| Carrier | 89 | 14 | 1.1 | 13.0 (7.1–21.7) | 97 | 12 | 0.9 | 13.0 (6.7–22.7) | 2 | 1.9 | 1.0 |
| Possible carrier | 464 | 17 | 11.4 | 1.5 (0.9–2.4) | 502 | 16 | 11.6 | 1.4 (0.8–2.2) | 21 | 24.2 | 0.9 |
| Probable non-carrier | 801 | 24 | 22.9 | 1.0 (0.7–1.6) | 839 | 19 | 22.9 | 0.8 (0.5–1.3) | 29 | 48.4 | 0.6 |

[a]95% confidence interval for ratio *O/E*.

**Table III** Non-ocular neoplasms in 'carrier' parents included in the analyses of cancer registrations and death certificates

| | *Non-ocular neoplasm* | | |
|---|---|---|---|
| *Retinoblastoma laterality* | *Diagnosis* | *Cancer registration age (years)* | *Death certificate age (years)* |
| *Fathers* | | | |
| Unilateral | Cancer lung | 45 | 45 |
| Bilateral | Cancer oesophagus | 62 | 64 |
| Bilateral | Cancer lung | 34 | 35 |
| Bilateral | Glioma | 44 | 44 |
| Bilateral | Glioma | 34 | 34 |
| Unilateral[a] | Cancer lung | – | 48 |
| *Mothers* | | | |
| Unilateral | Cancer intestine | 43 | 44 |
| Bilateral | Cancer lung | 41 | 41 |
| Unilateral | Leiomyosarcoma | 41 | 45 |
| Bilateral | Melanoma | 42 | 42 |
| Bilateral | Cancer bladder | 45 | 46 |
| Bilateral | Cancer ovary | 30 | 32 |
| Unilateral[b] | Cancer breast | 41 | – |
| Bilateral[b] | Melanoma | 31 | – |
| Unilateral[b] | Meningioma | 40 | – |

[a]Included in death analysis, not in cancer registration analysis; [b]Included in cancer registration analysis, not in death analysis.

**Table IV** Observed and expected numbers of cancer registrations and deaths from cancer among parents who are carriers of retinoblastoma, divided by type of neoplasm

| | *Cancer registrations* | | | *Deaths from cancer* | | |
|---|---|---|---|---|---|---|
| *Type of neoplasm* | *Observed* | *Expected* | *O/E* | *Observed* | *Expected* | *O/E* |
| Respiratory (lung and bronchus) | 3 | 0.18 | 16.4 | 4 | 0.20 | 19.7 |
| Melanoma | 2 | 0.03[a] | 73.3 | 1 | 0.02 | 55.6 |
| Breast cancer | 1 | 0.25 | 4.1 | 0 | 0.14 | – |
| Bladder cancer | 1 | 0.03 | 28.8 | 1 | 0.01 | 71.7 |
| Brain tumours (including non-malignant) | 3 | 0.04 | 73.7 | 2 | 0.05 | 37.9 |

For each group, with the exception of breast cancer, the observed numbers are significantly greater than the expected numbers ($P < 0.05$ in each case).

[a]The use of 1968–78 registration data leads to some underestimate in the expected number for melanoma.

Table V lists the 16 parents among the carrier and possible carrier groups who have had to be excluded, and the reasons for exclusion.

In view of the association between retinoblastoma and melanoma it should be noted that in addition to the two mothers who were carriers and developed malignant melanoma and were included in the analysis, two other carrier mothers also developed malignant melanoma. One was successfully treated before the birth of the index child and the second mother developed melanoma after the end of the study date.

Another tumour which has been associated with retinoblastoma is bladder cancer. One mother who was a carrier of retinoblastoma died of bladder cancer and has been included in the analysis. In addition a mother who was a possible carrier died of bladder cancer after the end of the study date, and a father included in the analysis who was said to have had a congenital cataract, but is not included among the carriers of retinoblastoma for lack of precise information, also died of bladder cancer.

*Cancer in grandparents, uncles and aunts of retinoblastoma patients*

Three hundred and sixteen families have been included in the study of cancers in grandparents, uncles and aunts, and in 64 of these families there has been more than one case of retinoblastoma. A total of 2,663 of these relatives were identified in the course of the interviews and follow-up information obtained up to the date of the interview for nearly 99% of them. A small number of cases are excluded from the analysis because the information available for them lies outside the appropriate period of risk.

Table VI gives the ratios of observed to expected numbers of reported cancers, deaths from cancer and deaths from causes other than cancer for grandparents, uncles and aunts

**Table V** Non-ocular neoplasms in 'carrier' and 'possible carrier' parents excluded from the analyses of cancer registrations and death certificates

| *Diagnosis* | *Age at diagnosis (years)* | *Reason for exclusion* |
|---|---|---|
| *Mothers* | | |
| *Carriers* | | |
| Squamous cell carcinoma, (skin), died carcinoma lung | 52 | 1st: diagnostic type not included<br>2nd: died after end of study date |
| Carcinoma lung and carcinoma of breast | 39 | After end of study date |
| Melanoma | 39 | After end of study date |
| Melanoma | 18 | Before birth of index child |
| Fibrosarcoma | 18 | Before birth of index child |
| *Possible carriers* | | |
| Cancer of bladder | 45 | After end of study date |
| Carcinoma-*in-situ* cervix | 24 | Diagnostic type not included |
| Cancer of breast | 42 | After end of study date |
| Squamous cell carcinoma cervix | 27 | Diagnostic type not included |
| *Fathers* | | |
| *Possible carriers* | | |
| Basal cell carcinoma | 62 | After end of study date |
| Lymphoma | 47 | After end of study date |
| Cancer rectum | 55 | After end of study date |
| Cancer colon | 52 | After end of study date |
| Carcinoma lung | 65 | After end of study date |
| Carcinoma lung | 62 | After end of study date |
| Carcinoma stomach | 78 | After end of study date |

**Table VI** Observed and expected numbers of cancers and deaths from other causes among relatives of retinoblastoma patients

| | | *Reported cancer* | | | *Deaths from cancer* | | | *Deaths from other causes* | | |
|---|---|---|---|---|---|---|---|---|---|---|
| | *Total in group* | *Observed* | *Expected* | *O/E* | *Observed* | *Expected* | *O/E* | *Observed* | *Expected* | *O/E* |
| *Grandparents* | | | | | | | | | | |
| Group 1 | 15 | 9 | 1.1 | 8.3 | 9 | 0.8 | 11.3 | 1 | 2.1 | 0.5 |
| 2 | 10 | 3 | 1.2 | 2.4 | 2 | 0.9 | 2.1 | 2 | 2.4 | 0.8 |
| 3 | 74 | 15 | 8.9 | 1.7 | 12 | 6.5 | 1.8 | 15 | 17.1 | 0.9 |
| 4 | 10 | 5 | 1.2 | 4.1 | 4 | 1.0 | 4.0 | 3 | 3.2 | 0.9 |
| 5 | 511 | 50 | 61.1 | 0.8 | 43 | 45.4 | 0.9 | 116 | 115.4 | 1.0 |
| 6 | 453 | 35 | 57.5 | 0.6 | 26 | 43.2 | 0.6 | 103 | 106.6 | 1.0 |
| 7 | 123 | 8 | 17.0 | 0.5 | 4 | 13.0 | 0.3 | 24 | 32.8 | 0.7 |
| Total | 1,196 | 125 | 148.0 | 0.8 | 100 | 110.8 | 0.9 | 264 | 279.5 | 0.9 |
| *Aunts and uncles* | | | | | | | | | | |
| Group 1 | 15 | 2 | 0.2 | 11.1 | 2 | 0.1 | 22.2 | 1 | 0.3 | 3.2 |
| 2 | 18 | – | 0.2 | – | – | 0.1 | – | 1 | 0.5 | 2.1 |
| 3 | 65 | 2 | 1.1 | 1.8 | 1 | 0.7 | 1.5 | 1 | 2.0 | 0.5 |
| 4 | 18 | – | 0.8 | – | – | 0.5 | – | – | 1.0 | – |
| 5 | 653 | 7 | 9.9 | 0.7 | 6 | 5.3 | 1.1 | 12 | 14.5 | 0.8 |
| 6 | 531 | 2 | 6.4 | 0.3 | – | 3.4 | – | 7 | 10.0 | 0.7 |
| 7 | 120 | – | 1.6 | – | – | 0.8 | – | 3 | 2.5 | 1.2 |
| Total | 1,420 | 13 | 20.2 | 0.6 | 9 | 10.8 | 0.8 | 25 | 30.7 | 0.8 |

**Table VII** Observed and expected numbers of cancers and deaths from other causes among 'carrier', 'possible carrier' and other relatives of retinoblastoma patients

| | | *Reported cancer* | | | *Deaths from cancer* | | | *Deaths from other causes* | | |
|---|---|---|---|---|---|---|---|---|---|---|
| | *Total in group* | *Observed* | *Expected* | *O/E (95% CI)*[a] | *Observed* | *Expected* | *O/E (95% CI)*[a] | *Observed* | *Expected* | *O/E* |
| *Grandparents* | | | | | | | | | | |
| Carriers | 15 | 9 | 1.1 | 8.3 (3.8–15.8) | 9 | 0.8 | 11.2 (5.1–21.2) | 1 | 2.1 | 0.5 |
| Possible carriers | 94 | 23 | 11.4 | 2.0 (1.3–3.0) | 18 | 8.4 | 2.1 (1.3–3.4) | 20 | 22.7 | 0.9 |
| Probable non-carriers | 1,087 | 93 | 135.5 | 0.7 (0.6–0.8) | 73 | 101.6 | 0.7 (0.6–0.9) | 243 | 254.8 | 1.0 |
| *Aunts and uncles* | | | | | | | | | | |
| Carriers | 15 | 2 | 0.2 | 11.1 (1.3–40.1) | 2 | 0.1 | 22.2 (2.7–80.2) | 1 | 0.3 | 3.3 |
| Possible carriers | 101 | 2 | 2.1 | 1.0 (0.1–2.5) | 1 | 1.3 | 0.8 (0.0–4.4) | 2 | 3.4 | 0.6 |
| Probable non-carriers | 1,304 | 9 | 17.9 | 0.5 (0.2–1.0) | 6 | 9.5 | 0.6 (0.2–1.4) | 22 | 26.9 | 0.8 |

[a]95% confidence interval for ratio O/E.

of the index child, subdivded according to the likelihood of being a retinoblastoma carrier as explained in the **Methods** section. As with the parents of retinoblastoma patients the relatives have then been grouped into three categories: carriers, possible carriers and probable non-carriers. Ratios of observed to expected numbers are given in Table VII. The non-ocular neoplasms among relatives who are carriers of the retinoblastoma gene are listed in Table VIII. Grandparents who were carriers have a risk eight times as great as expected of developing cancer, and 11 times as great as expected of dying from cancer (the 95% confidence limits for these ratios of observed to expected numbers are 3.8 and 15.8, 5.1 and 21.2). Grandparents who were possible carriers have twice the expected risk of developing cancer and dying from cancer (95% confidence limits 1.3 and 3.0, 1.3 and 3.4). The small numbers of observed cancers and deaths from cancer among uncles and aunts who were carriers or possible carriers give wide confidence limits for the ratios, though there is a significant excess of cancer in the carrier group. The deficit of cancers observed among the relatives who are probable non-carriers may be a result of incomplete reporting of cancers and deaths or of misclassification of causes of death in cases where medical records or death certificates were not obtained.

The great majority of children in the hospital-based pedigree study were accompanied by their mothers and a comparison of the information provided about maternal and paternal relatives disclosed a generally higher reported incidence of cancers and deaths among maternal than among paternal relatives, particularly among the uncles and aunts. However, the general conclusions are unaffected by this slight bias.

There may have been a certain degree of under-reporting of events, particularly among paternal relatives, and it is possible that the excess risks are higher than quoted here.

**Table VIII** Non-ocular neoplasms in 'carrier' relatives included in the analyses of cancers and deaths from cancer

| | *Non-ocular neoplasm* | |
|---|---|---|
| *Retinoblastoma laterality* | *Diagnosis* | *Age at diagnosis (years)* |
| *Grandfathers* | | |
| No record | Cancer bladder | 63 |
| Unaffected[a] | Cancer colon | 77 |
| Unilateral | Cancer lung | 45 |
| Bilateral | Cancer lung | 52 |
| Bilateral | Spindle cell sarcoma | 47 |
| Unaffected | Cancer lung | 44 |
| *Grandmothers* | | |
| Bilateral | Cancer lung | 41 |
| Unaffected | Cancer lung | 62 |
| Unilateral | Cancer uterus | 43 |
| *Uncle* | | |
| Unilateral[a] | Cancer lung | 56 |
| *Aunt* | | |
| Bilateral[a] | Cancer ovary | 33 |

[a]Members of the same family.

### *Summary of results for all relatives*

A high risk of developing non-ocular neoplasms has been reported in younger survivors from retinoblastoma and the results from this study show that this increased risk continues in older survivors who have become parents and grandparents.

Combining the results of the two separate analyses, we find that in a total of 117 relatives of retinoblastoma patients who were themselves carriers of the mutated retinoblastoma gene there were 23 cases of non-ocular cancer compared with an expected number of 2.3, a relative risk of 9.9 (95% confidence limits of 6.3 and 14.9). (The total of 117 relatives is smaller than the totals in Tables II and VI because two individuals appear twice in the analysis: one as both mother and grandmother, the second as father and grandfather. This does not of course affect the results of the separate analyses.) In a total of 25 deaths from all causes among relatives, 21 deaths were from non-ocular cancer. The expected number was 1.8, giving a relative risk of 11.6 (95% confidence limits of 7.2 and 17.7).

Among the relatives who were *possible* carriers, there were 42 cases of non-ocular cancer compared with an expected number of 24.8, giving a relative risk of 1.7. Thirty-five relatives died of cancer as compared with an expected number of 21.3, a relative risk of 1.6. This reflects the fact that this group is a mixture of individuals who are in fact carriers, and who have an excess risk of developing non-ocular cancer, together with others who do not have the Rb− gene and therefore presumably have no increased risk.

### Discussion

There have been several studies of non-ocular cancer in relatives of retinoblastoma patients. Strong *et al.* (1984), in a study of 80 families, found a significant excess of cancer deaths in relatives under the age of 55 and in fathers of the bilateral retinoblastoma probands, and a modest overall cancer excess in the total group of relatives of hereditary cases. Der Kinderen *et al.* (1988) found four non-ocular cancers compared with 1.9 expected, among 24 parents of retinoblastoma patients who were themselves carriers of the retinoblastoma gene. Two hundred and six unaffected parents of patients with hereditary retinoblastoma were also followed up and among this group 23 cancers developed: the fathers were found to have a relative risk of 8.3 for pancreatic cancer compared with the general population; no increased risk was found for other non-ocular cancers. Winther *et al.* (1988) followed up 267 parents of retinoblastoma survivors, and, after excluding five who had been treated for retinoblastoma, observed 14 cases of non-ocular cancer, a relative risk of 0.9. Three of the cancers were malignant melanomas. The parents were not subdivided according to whether their children had unilateral or bilateral retinoblastoma.

It is well documented that retinoblastoma survivors are at a high risk of developing second primary neoplasms (Abramson *et al.*, 1984; Draper *et al.*, 1986; der Kinderen *et al.*, 1988; Strong *et al.*, 1984). In adolescence the risk is particularly high for osteosarcomas. The results of this study give further evidence that the risk persists through the lives of the survivors.

These previous studies have mentioned melanoma as being a second tumour particularly associated with retinoblastoma, and the four carrier mothers in this study who developed malignant melanoma between the ages of 18 and 42 bear out this finding. Two grandmothers of retinoblastoma patients were also diagnosed as having malignant melanoma, they were not known to have had retinoblastoma.

There is a high ratio of observed to expected numbers of deaths from cancer of the lung (15.4) among relatives who are carriers of the mutated retinoblastoma gene. The total of eight patients in this analysis of deaths from lung cancer included four with small cell or oat cell carcinoma and one with undifferentiated adenocarcinoma. The tumour histology of the other three patients is not known. Two carrier mothers excluded from the analysis because their second tumours developed after the end of the study date also died of lung cancer: one small cell carcinoma, the other oat cell carcinoma. (One of these patients had a carcinoma of the breast in addition to the lung cancer.) Strong *et al.* (1984) observed a significant excess of lung cancer in relatives under 55. Harbour *et al.* (1988) and Yokota *et al.* (1988) suggested that inactivation of the Rb gene may be involved in the development of lung cancers, particularly small cell carcinoma.

Several studies have suggested an association between retinoblastoma and bladder cancer. Der Kinderen *et al.* (1988) observed two transitional cell carcinomas of the bladder among 24 carrier parents. Tarkkanen & Karjalainen (1984) noted two bladder carcinomas among 19 relatives of patients with bilateral retinoblastoma. Two relatives in this study who were carriers and six other relatives of children with bilateral tumours also died of bladder cancer. Der Kinderen *et al.* (1988) also found three pancreatic tumours among unaffected fathers of children with hereditary retinoblastoma. In this study we noted that two unaffected parents and two unaffected grandparents of children with bilateral tumours died of cancer of the pancreas.

Among parents of retinoblastoma patients included in the cancer registration analysis there were 89 carriers of the retinoblastoma gene, of whom 70 had themselves had retinoblastoma. Non-ocular cancer developed in 20 of these survivors although the analyses of risks can only take account of 15, for reasons explained above. It is interesting that among the 19 carrier parents who had not themselves had retinoblastoma no non-ocular cancers have so far been recorded. They are known to be carriers because in addition to having a child with retinoblastoma, either siblings of their own, children of siblings or relatives in a previous generation were affected by retinoblastoma. In one family, three unaffected brothers all had children with retinoblastoma. These unaffected carriers appear to be inherently less liable to the further genetic changes which lead to the development of both retinoblastoma and subsequent non-ocular cancer. Matsunaga (1979) postulated a host resistance model whereby unaffected carriers are inherently resistant to tumour formation. However, it should be noted that among four grandparents who were certainly carriers but not reported to have had retinoblastoma themselves, three subsequently developed non-ocular cancer.

It is clear that inheritance of the Rb− gene confers susceptibility to other tumours in later life. This is shown conclusively in this study among the known carriers of the gene.

The overall excess of cancer deaths of 1.6 among relatives who were possible carriers of the retinoblastoma gene agrees with the findings of Strong *et al.* (1984) and can be attributed to the fact that a proportion of these relatives are also in fact carriers of the gene and have a raised likelihood of developing cancer in later life, though the risk may be less than that for carriers who are themselves affected by retinoblastoma.

When all carriers and possible carriers of the retinoblastoma gene are excluded from the analysis there is a lower than average mortality among both parents and other relatives. We think this may be partly accounted for by under-reporting of events, both deaths and cancers, and, in particular, by the implicit assumption that relatives for whom neither of these events were reported at the interview were alive and well until that time. As a consequence the relative risks reported in this paper are likely to be underestimated. An alternative explanation that may partly account for the deficit of deaths among parents is, by analogy with the 'healthy worker' effect, that members of the population who become parents are a selected group having a lower mortality than others in their age group – a 'healthy parent' effect.

We thank the consultants and general practitioners who provided information on which this paper is based. We are especially grateful to the parents of children with retinoblastoma who agreed to be interviewed and to give information about their families. The Office of Population Censuses and Surveys, the Information and Statistics Division of the Common Services Agency of the Scottish Health Service, the Registrar General for Scotland and regional cancer registries provided notifications of cancers and deaths. We are grateful to them and also to the National Health Service Central Registers in Southport and Edinburgh for 'flagging' patients and notifying subsequent cancers and deaths. We would like to thank Dr J.E. Kingston and Mr J. Hungerford of St Bartholomew's and Moorfields Hospitals for advice and for allowing access to hospital records, and Ms H. Smith who carried out the extensive computing work required for this study. The Childhood Cancer Research Group is supported by grants from the Department of Health and Social Security and the Scottish Home and Health Department. Dr Jay receives a grant from the Cancer Research Campaign.

### References

ABRAMSON, D.H., ELLSWORTH, R.M., KITCHIN, F.D. & TUNG, G. (1984). Second non-ocular tumours in retinoblastoma survivors. *Ophthalmology*, **91**, 1351.

BONAITI-PELLIE, C. & BRIARD-GUILLEMOT, M.L. (1980). Excess of cancer deaths in grandparents of patients with retinoblastoma. *J. Med. Genet.*, **17**, 95.

COLEMAN, M., DOUGLAS, A., HERMON, C. & PETO, J. (1986). Cohort study analysis with a Fortran computer program. *Int. J. Epidemiol.*, **15**, 134.

DER KINDEREN, D.J., KOTEN, J.W., NAGELKERKE, N.J.D., TAN, K.E.W.P., BEEMER, F.A. & DEN OTTER, W. (1988). Non-ocular cancer in patients with hereditary retinoblastoma and their relatives. *Int. J. Cancer*, **41**, 499.

DRAPER, G.J., SANDERS, B.M. & KINGSTON, J.E. (1986). Second primary neoplasms in patients with retinoblastoma. *Br. J. Cancer*, **53**, 661.

FEDRICK, J. & BALDWIN, J.A. (1978). Incidence of cancer in relatives of children with retinoblastoma. *Br. Med. J.*, **i**, 83.

FRIEND, S.H., BERNARDS, R., ROGELJ, S. and 4 others (1986). A human DNA segment with properties of the gene that predisposes to retinoblastoma and osteosarcoma. *Nature*, **323**, 643.

GORDON, H. (1974). Family studies in retinoblastoma. *Med. Genet. Today*, **10**, 185.

HARBOUR, J.W., LAI, S., WHANG-PENG, J., GAZDAR, A.F., MINNA, J.D. & KAYE, F.J. (1988). Abnormalities in structure and expression of the human retinoblastoma gene in SCLC. *Science*, **241**, 353.

KNUDSON, A.G. (1978). Retinoblastoma: a prototypic hereditary neoplasm. *Semin. Oncol.*, **5**, 57.

LEE, E.Y.-H.P., TO, H., SHEW, J.-Y., BOOKSTEIN, R., SCULLY, P. & LEE, W.-H. (1988). Inactivation of the retinoblastoma susceptibility gene in human breast cancers. *Science*, **241**, 218.

MATSUNAGA, E. (1978). Hereditary retinoblastoma: delayed mutation or host resistance. *Am. J. Hum. Genet.*, **30**, 406.

MURPHREE, A.L. & BENEDICT, W.F. (1984). Retinoblastoma: clues to human oncogenesis. *Science*, **223**, 1028.

STRONG, L.C., HERSON, J., HAAS, C. and 4 others (1984). Cancer mortality in relatives of retinoblastoma patients. *J. Natl Cancer Inst.*, **73**, 303.

TARKKANEN, A. & KARJALAINEN, K. (1984). Excess of cancer deaths in close relatives of patients with bilateral retinoblastoma. *Ophthalmologica*, **189**, 143.

WINTHER, J., OLSEN, J.H. & DE NULLY BROWN, P. (1988). Risk of non-ocular cancer among retinoblastoma patients and their parents. *Cancer*, **62**, 1459.

YOKOTA, J., AKIYAMA, T., FUNG, Y.-K.T. and 8 others (1988). Altered expression of the retinoblastoma (RB) gene in small-cell carcinoma of the lung. *Oncogene*, **3**, 471.

**Appendix**

Likelihood that grandparent, uncle or aunt of index child was a carrier of the mutation at the RB locus.

| *Criteria, in addition to index child having retinoblastoma (RBL)* | | |
|---|---|---|
| 1. Carrier | Grandparent | (*a*) Had RBL, treated or spontaneously regressed<br>(*b*) Any relative in the same or previous generation had RBL or was a carrier of RBL<br>(*c*) Children by two different spouses had RBL |
| | Uncle/aunt | (*a*) Had RBL, treated or spontaneously regressed<br>(*b*) Child or grandchild had RBL |
| 2. Possible carrier (where spouse did not have RBL) 50% likelihood | Grandparent | Child (other than parent of index child) had RBL or was carrier of RBL |
| | Uncle/aunt | (*a*) Parent had RBL or was a carrier of RBL<br>(*b*) Sib (other than parent of index child) had RBL or was carrier of RBL |
| 3. Possible carrier (where spouse did not have RBL) | Grandparent | Only one child had RBL or was carrier of RBL |
| | Uncle/aunt | Only one sib had RBL or was carrier of RBL |
| 4. Possible carrier (where spouse did not have RBL) | Grandparent | Two or more grandchildren in one family had RBL (one of these was the index child) |
| | Uncle/aunt | Two or more nephews or nieces in one family had RBL (one of these was the index child) |
| 5. Probably not a carrier | Grandparent | Only index child had RBL and tumours were bilateral |
| | Uncle/aunt | Only index child had RBL and tumours were bilateral |
| 6. Probably not a carrier | Grandparent | Only index child had RBL and tumour was unilateral |
| | Uncle/aunt | Only index child had RBL and tumour was unilateral |
| 7. Not a carrier | Grandparent | Family history of RBL on other side of family |
| | Uncle/aunt | Family history of RBL on other side of family |